# Editorial Comment on “Two cases of CRPC with *BRCA* mutation treated by olaparib after favorable response to cisplatin”

**DOI:** 10.1002/iju5.12557

**Published:** 2022-11-08

**Authors:** Koji Hatano

**Affiliations:** ^1^ Department of Urology Osaka University Graduate School of Medicine Suita Japan

Mutations associated with DNA repair are found in about one‐quarter of prostate cancers, with *BRCA2* being the most frequently affected genes and with *BRCA1/2* mutations occurring both at the somatic and germline levels. Metastatic castration‐resistant prostate cancer (mCRPC) with *BRCA1/2* mutations is preferentially sensitive to poly (adenosine diphosphate–ribose) polymerase (PARP) inhibitors via a synthetic lethal mechanism. In addition, emerging evidence suggests that patients with *BRCA2*‐related CRPC responded to platinum‐based chemotherapy at a high rate. In this case report, Miyazawa *et al*. reported two cases of mCRPC with *BRCA* mutations that responded well to cisplatin followed by olaparib.^1^ Recently, Slootbeek *et al*. reported four cases of mCRPC with *BRCA2* mutations that responded to both platinum‐based chemotherapy and subsequent PARP inhibitors.^2^ Mota *et al*. evaluated the efficacy of platinum‐based chemotherapy after PARP inhibitor treatment in mCRPC patients with mutations in DNA damage repair genes. Of the 6 patients with *BRCA1/2* mutations, 3/5 evaluable patients had a radiological partial response or stable disease and 2/5 had a PSA50 response.^3^ These results provide insights into cross‐resistance between PARP inhibitors and platinum chemotherapy in patients with *BRCA*‐mutated mCRPC.

The two cases presented in this case report were treatment‐induced neuroendocrine prostate cancer (NEPC), which is generally considered to have a poor prognosis.^1^ NEPC is an aggressive variant of prostate cancer, which may occur de novo or in patients who have previously received hormone therapy. Genomic features of NEPC are associated with reduced AR signaling and concurrent loss of *RB1* and *TP53*.^4^ Platinum‐based chemotherapy regimens, such as the combination of cisplatin and etoposide, are widely used in NEPC patients. In men with NEPC who have responded to platinum‐based chemotherapy, genetic testing to detect PARP inhibitor candidates may be worth considering.

## Funding information

This research was supported by the Japan Society for the Promotion of Science KAKENHI (grant number 22K09447).

## Conflict of interest

KH has received grants/research supports from Astellas Pharma Inc.
